# Comprehensive analysis of M2 macrophage-derived exosomes facilitating osteogenic differentiation of human periodontal ligament stem cells

**DOI:** 10.1186/s12903-022-02682-5

**Published:** 2022-12-27

**Authors:** Xian-min Liao, Zheng Guan, Zhen-jin Yang, Li-ya Ma, Ying-juan Dai, Cun Liang, Jiang-tian Hu

**Affiliations:** 1 Department of Orthodontics, Hospital of Stomatology, Kunming Medical University/Yunnan Stomatology Hospital, Building C, Hecheng International, No. 1088 Middle Haiyuan Road, Kunming, 650106 Yunnan Province China; 2 Stomatology Center, the First People’s Hospital of Yunnan, Kunming, China; 3 Biomedical Research Center, Affiliated Calmette Hospital of Kunming Medical University/the First Hospital of Kunming, Kunming, China

**Keywords:** Orthodontic tooth movement, Periodontal ligament stem cells, Macrophage polarization, Exosome, microRNA

## Abstract

**Background:**

The role of periodontal ligament stem cells (PDLSCs) and macrophage polarization in periodontal tissue regeneration and bone remodeling during orthodontic tooth movement (OTM) has been well documented. Nevertheless, the interactions between macrophages and PDLSCs in OTM remain to be investigated. Consequently, the present study was proposed to explore the effect of different polarization states of macrophages on the osteogenic differentiation of PDLSCs.

**Methods:**

After M0, M1 and M2 macrophage-derived exosomes (M0-exo, M1-exo and M2-exo) treatment of primary cultured human PDLSCs, respectively, mineralized nodules were observed by Alizarin red S staining, and the expression of ALP and OCN mRNA and protein were detected by RT-qPCR and Western blotting, correspondingly. Identification of differentially expressed microRNAs (DE-miRNA) in M0-exo and M2-exo by miRNA microarray, and GO and KEGG enrichment analysis of DE-miRNA targets, and construction of protein–protein interaction networks.

**Results:**

M2-exo augmented mineralized nodule formation and upregulated ALP and OCN expression in PDLSCs, while M0-exo had no significant effect. Compared to M0-exo, a total of 52 DE-miRNAs were identified in M2-exo. The expression of hsa-miR-6507-3p, hsa-miR-4731-3p, hsa-miR-4728-3p, hsa-miR-3614-5p and hsa-miR-6785-3p was significantly down-regulated, and the expression of hsa-miR-6085, hsa-miR-4800-5p, hsa-miR-4778-5p, hsa-miR-6780b-5p and hsa-miR-1227-5p was significantly up-regulated in M2-exo compared to M0-exo. GO and KEGG enrichment analysis revealed that the downstream targets of DE-miRNAs were mainly involved in the differentiation and migration of multiple cells.

**Conclusions:**

In summary, we have indicated for the first time that M2-exo can promote osteogenic differentiation of human PDLSCs, and have revealed the functions and pathways involved in the DE-miRNAs of M0-exo and M2-exo and their downstream targets.

**Supplementary Information:**

The online version contains supplementary material available at 10.1186/s12903-022-02682-5.

## Introduction

Orthodontic tooth movement (OTM) is a process of bone remodeling occurring in periodontal tissues through a series of physiological reactions, with bone remodeling on the tension side of the bone and bone resorption on the pressure side, ultimately achieving tooth movement [1, 2]. The periodontium connects the cementum to the alveolar bone through the type I fibers, which maintain the nutritional supply of the orthodontic teeth, and transmit and absorb mechanical stresses [3]. Periodontal ligament stem cells (PDLSCs), a critical cell in periodontal tissue engineering, were first identified by Seo et al. [4] when isolated and cultured from periodontal tissue of third molars. PDLSCs have been shown to have a positive effect on OTM through their multipotential differentiation capacity towards osteogenic, lipogenic and chondrogenic lineages similar to other mesenchymal stem cells, forming new periodontal support tissues to reconstruct the attachment relationship between cementum and alveolar bone [4, 5]. Consequently, maintaining and promoting the properties of PDLSCs is essential for OTM.

Exosomes are nanoscale vesicles that can be secreted by most cells, with a diameter of about 30–150 nm, and are widely present in various body fluids in the human body. Exosomes carry a variety of biologically active substances, such as proteins, lipids, DNA and RNA, which play an important role in intercellular communication and signal transduction, and are involved in the regulation of biological processes, including the immune response, cell proliferation and differentiation. [6, 7]. Previous studies have demonstrated that human exfoliated deciduous teeth (SHED)-derived exosomes can promote osteogenic differentiation of PDLSCs through activation of Wnt and BMP signaling pathways [8]. Bone marrow mesenchymal stem cells (BMSCs)-derived exosomes enhance migration, proliferation and differentiation of PDLSCs to promote periodontal regeneration [9]. Mechanical strain-induced osteocyte-derived exosomes inhibit PTEN/AKT signaling pathway via miR-181b-5p to promote proliferation and osteogenic differentiation of human PDLSCs (hPDLSCs) by BMP2/Runx2 [10]. It is indicated that multiple sources of exosomes contribute to the osteogenic differentiation of PDLSCs, and it is positive to deeply explore the effect of exosomes on PDLSCs for OTM treatment.

Macrophages, as one of the main immune cells in the periodontal microenvironment, are involved in OTM. Macrophage polarization exists mainly in the state of classically activated macrophages (M1 macrophages) and alternatively activated macrophages (M2 macrophages) [11, 12]. Osteoclasts, which play a major role on the pressure side of the OTM process, are derived from monocytes/macrophages. Earlier studies have shown the presence of M1 macrophages on the stress side of OTM and that the proportion is significantly increased [13, 14]. Notably, the interaction between macrophages and PDLSCs via exosomes may be indispensable for repair and regeneration of periodontal tissue and OTM. Previous studies have revealed that PDLSCs induce macrophages towards the M2 phenotype, and contribute to the enhancement of periodontal regeneration during the early stages of tissue repair [15]. Liu et al. [16] also showed that PDLSCs promote M2 polarization in macrophages via the JNK pathway. LPS-stimulated PDLSCs induce M1 polarization of macrophages via extracellular vesicles, which is detrimental to osteogenic differentiation [17]. He et al. [18] indicated that PDLSCs produce H_2_S in response to mechanical stimulation to promote macrophage polarization to the M1 phenotype, which contributes to bone remodeling and OTM. It is suggested that the regulation of macrophage polarization by PDLSCs is an important pathway for periodontal tissue regeneration and OTM. However, it remains to be investigated whether exosomes derived from different polarization states of macrophages have an effect on the osteogenic differentiation of PDLSCs.

Based on the above studies, we hypothesized that exosomes derived from different polarization states of macrophages are involved in regulating the osteogenic differentiation process of PDLSCs. Furthermore, we analyzed the differentially expressed microRNAs (DE-miRNA) in M0 macrophage-derived exosomes (M0-exo) and M2 macrophage-derived exosomes (M2-exo) by miRNA microarray. It aimed to elucidate the interaction mechanism between macrophages and PDLSCs in OTM, and to disclose the key factors.

## Materials and method

### Induction and identification of macrophage polarization

THP-1 cells and complete medium were purchased from National Collection of Authenticated Cell Cultures (China; Cat. No.: TCHu 57). THP-1 cells were inoculated in complete medium and cultured in incubator conditions of 37 °C, 5% CO_2_, and 90% humidity. THP-1 cells (1 × 10^6^ cells /mL) were inoculated in 6-well plates and M0 macrophages were induced by stimulation with 100 ng/ml PMA (Solarbio, China) for 6 h. M0 macrophages were incubated with 20 ng/ml IFN-γ and 100 ng/ml LPS (Solarbio) for 48 h to induce M1 macrophages. M0 macrophages were incubated with 20 ng/ml IL-4 and 20 ng/ml IL-13 (Solarbio) for 48 h to induce M2 macrophages. Macrophages were collected, and CD68 and CD80, CD16 and CD86, CD163 and CD206 (BD Biosciences, San Jose, CA, USA) were detected using a BD-FACSAria™ Fusion flow cytometer (BD Biosciences, San Jose, CA, USA) to identify M0, M1 and M2 macrophages as described previously, respectively [19, 20].

### Isolation and identification of macrophage-derived exosomes

Briefly, after induction of THP-1 cells (1 × 10^6^ cells /mL) into M0, M1 and M2 macrophages, RPMI-1640 complete medium was continued for 24 h and then replaced with serum-free RPMI-1640 medium for 24 h. M0 macrophage-derived exosomes (M0-exo), M1 macrophage-derived exosomes (M1-exo) and M2 macrophage-derived exosomes (M2-exo) were prepared by differential centrifugation. Briefly, the supernatants of M0, M1 and M2 macrophages were collected and centrifuged at 300 g for 10 min to remove cells, at 2000 g for 10 min to remove dead cells and at 10,000 g for 30 min with 4℃ to remove cell debris. Subsequently, the supernatant was transferred to an ultracentrifuge tube and centrifuged at 100,000 g for 90 min with 4℃ to precipitate exosomes. Finally, the exosomes were resuspended in pre-cooled PBS and centrifuged again at 100,000 g for 90 min with 4℃ to obtain exosomes. Moreover, M0-exo, M1-exo and M2-exo were quantified using a spectrophotometer at 405 nm by referring to instructions of EXOCET Exosome Quantitation Assay Kit (System Biosciences, San Francisco, CA, USA). The concentrations of M0-exo, M1-exo and M2-exo were 5.32 (OD_405nm_ = 0.09821), 5.96 (OD_405nm_ = 0.1059) and 4.36 (OD_405nm_ = 0.0867) μg/μl, respectively. According to the standard curve (y = 0.0012x + 0.0176) conversion, 1 × 10^6^ THP-1 cells can be separated approximately 6.718 × 10^8^ M0-exo, 7.358 × 10^8^ M1-exo and 5.758 × 10^8^ M2-exo, respectively. The morphological features of M0-exo, M1-exo and M2-exo were observed by Talos F200X G2 TEM transmission electron microscopy (TEM; Thermo Fisher Scientific, Waltham, MA, USA) as previously described [21]. Expression of exosome markers CD9, TSG101 and ALIX were detected by Western blotting for M0-exo, M1-exo and M2-exo and the corresponding supernatants. After identification, the lipid dyes PKH67 (Sigma-Aldrich) were used to label M0-exo, M1-exo and M2-exo by referring to the instructions.

### Isolation and identification of primary hPDLSCs

The hPDLSCs were separated from premolar teeth (15–30 years of age), which required extraction for orthodontic treatment. Inclusion criteria: (1) no systemic disease; (2) no history of smoking; (3) no history of long-term medication use; (4) healthy periodontal tissues. Exclusion criteria: (1) dental tissues with dental disease or inflammation; (2) patients refused to sign the informed consent form. All patient or guardian has signed an informed consent form, and the study has been approved by the Ethics Committee of Hospital of Stomatology, Kunming Medical University/Yunnan Stomatology Hospital (Approval Number: KYKQ2022MEC005). Primary culture of hPDLSCs was performed as described previously [22], and 3–5 generations of hPDLSCs were used for subsequent studies. CD4, CD90, CD34 and CD45 were detected by flow cytometry (BD Biosciences) as previously described [23, 24] to identify hPDLSCs. Lipogenic and osteogenic differentiation of hPDLSCs were induced as previously described [24], and were identified using oil red O staining and Alizarin red S staining, respectively.

After identification, osteogenic inducers (10–8 mol/L dexamethasone, 50 μg/ml vitamin C and 10 mmol/L sodium β-glycerophosphate) induced osteogenic differentiation of hPDLSCs, and hPDLSCs were treated with 10, 25, 50 and 100 μg/ml of M0-exo, M1-exo and M2-exo, respectively, for subsequent assays.

### Alizarin red S staining (ARS)

On day 21 of osteogenesis induction and exosome treatment, each group of hPDLSCs was fixed with 4% paraformaldehyde at room temperature for 30 min and incubated with 1% alizarin red staining solution (Solarbio) at 37 ℃ for 30 min. Mineralized nodules were observed and photographed under an inverted microscope. The calcified nodules were lysed in alizarin red-stained cells by adding 500 μl of 1% cetylpyridine chloride solution for 1 h. Subsequently, 200 μl of this solution was used to measure the absorbance of the calcified nodules by spectrophotometry at 562 nm.

### Western blotting assay

RIPA buffer (Beyotime, China) and ProteoPrep® Total Extraction Sample Kit (Sigma-Aldrich, St. Louis, MO, USA) were used to lyse hPDLSCs (osteogenesis induction and exosome treatment at d 7 and 21) and exosomes, respectively, to obtain total protein. The concentration of hPDLSCs and total exosomal proteins was measured using BCA Protein Assay Kit (Beyotime). Equal amounts of proteins were added on SDS-PAGE and electrophoresis was performed, followed by transfer to PVDF membrane. The membrane was blocked with 5% BSA for 2 h at room temperature and the exosome-loaded membranes were incubated overnight at 4 °C with rabbit monoclonal antibodies CD9 (1:1000; ab236630), TSG101 (1:3000; ab125011) and ALIX (1:1000; ab275377). The hPDLSCs samples were incubated overnight with rabbit polyclonal antibodies to ALP (1:1000; ab229126) and OCN (1:2000; ab93876). The membrane was washed 3 times with PBST and incubated with Goat Anti-Rabbit IgG H&L (HRP) (1:5000; abcam; ab205718) for 1 h at 37 °C. β-actin was used as an internal reference. The ECL kit (Beyotime) was used for development and the bands on the membrane were scanned and imaged by E-Gel Imager gel imaging system (Thermo Fisher Scientific). All the above antibodies were purchased from Abcam plc (Cambridge, MA, USA). The results were quantified using Image J software.

### RNA extraction and RT-PCR assay

Total RNA in hPDLSCs was extracted using Trizol reagent (Thermo Fisher Scientific). Extraction of miRNA from exosomes by Qiagen miRNeasy Mini Kit (QIAGEN, Duesseldorf, Germany). Total RNA was reverse-transcribed to cDNA according to the instructions of SuperScript First-Strand Synthesis System (Thermo Fisher Scientific), and RT-qPCR was performed by SYBR GreenMaster Mix (Thermo Fisher Scientific) to detect the expression of mRNA and miRNA. The primers for mRNA and miRNA are shown in Table 1. β-actin and U6 were used as internal references for mRNA and miRNA, respectively. The 2^−ΔΔCt^ method was used to calculate the relative expression levels of mRNA and miRNA.

**Table 1:** Primers used in RT-qPCR

Target	Forward primer (5′–3′)	Reverse primer (5′–3′)
ALP	CTGAAGCCTCCGTGGAACAT	TGACCACAAAGACTCAGCTC
OCN	CCCTTTCTCCTGTCCGGATG	GCTGAGCTCTAGGGGAGTCT
β-actin	GGCTCTTTTCCAGCCTTCCT	AATGCCAGGGTACATGGTGG
hsa-miR-6785-3p	GGAGGGCGTGGATGATGG	GGAAGGTGGGGCGATGTG
hsa-miR-6507-3p	GCCGAGCAAAGUCCUUCC	GUGUGGUCAGUACCAUGC
hsa-miR-4731-3p	GGTACAAGGTGCCTGTTGGA	CCTCAGGGTGAGGGATCTGA
hsa-miR-4728-3p	AGGATGATGCACTGTTGGGG	CTTGACTCCCCAGTGAACCC
hsa-miR-3614-5p	GTTCTGTCTTGGGCCACTTG	ACACCAAGATCTGAAGGCTAC
hsa-miR-6780b-5p	CAGCCTGGGGAAGGCTTG	AAAGGAGACAAGGGAGAGGC
hsa-miR-6085	TGGGCCCAGCTTTACATAGT	CCTTCAGCACCTTTTGCAACC
hsa-miR-4800-5p	GGAGTGGCCAGGAAGGAAG	CTGACAGGTAGGTGGACAGACG
hsa-miR-4778-5p	TCGGCAGGCUGUAAAGG	CAGTGCAGGGTCCGAGGTA
hsa-miR-1227-5p	GTGGTGGGCACTGCTGG	CTGGGGAAAAGGGTGGCA
U6	CCCTTCGGGGACATCCGATA	TTTGTGCGTGTCATCCTTGC

### MiRNA Microarray and bioinformatics analysis

MiRNA microarray was performed using Agilent Human miRNA Microarray Kit, Release 21.0, 8 × 60 K (Agilent, Santa Clara, CA, USA) by oebiotech (Shanghai, China). Briefly, total RNA in M0-exo and M2-exo was isolated using mirVana™ PARIS™ Kit (Invitrogen, Carlsbad, CA, USA). G2505C GeneChip Scanner (Agilent) for gene chip scanning. After scanning, the data was extracted by Feature Extraction software and the raw data were normalized by Genespring for subsequent data analysis. Bioinformatics analysis was also carried out by oebiotech. The screening threshold for DE-miRNA was absolute log_2_ FC ≥ 1 and *P* < 0.05. Downstream targeting of DE-miRNA was predicted through miRWalk and miRNADB databases. The threshold for GO [25] and KEGG [26] enrichment analysis of DE-miRNA targets was *P* < 0.05.

### Statistical analysis

All experiments were repeated at least three times, and data represent as mean ± standard deviation (SD). GraphPad Prism software (8.3.0 versions; GraphPad, San Diego, CA, USA) was used for statistical analysis and visualization. Student T test and One-Way ANOVA were used for comparison between two and multiple groups, respectively. *P* < 0.05 represents statistical significance.

## Results

### Isolation and identification of M0-exo, M1-exo and M2-exo

**Fig. 1:**
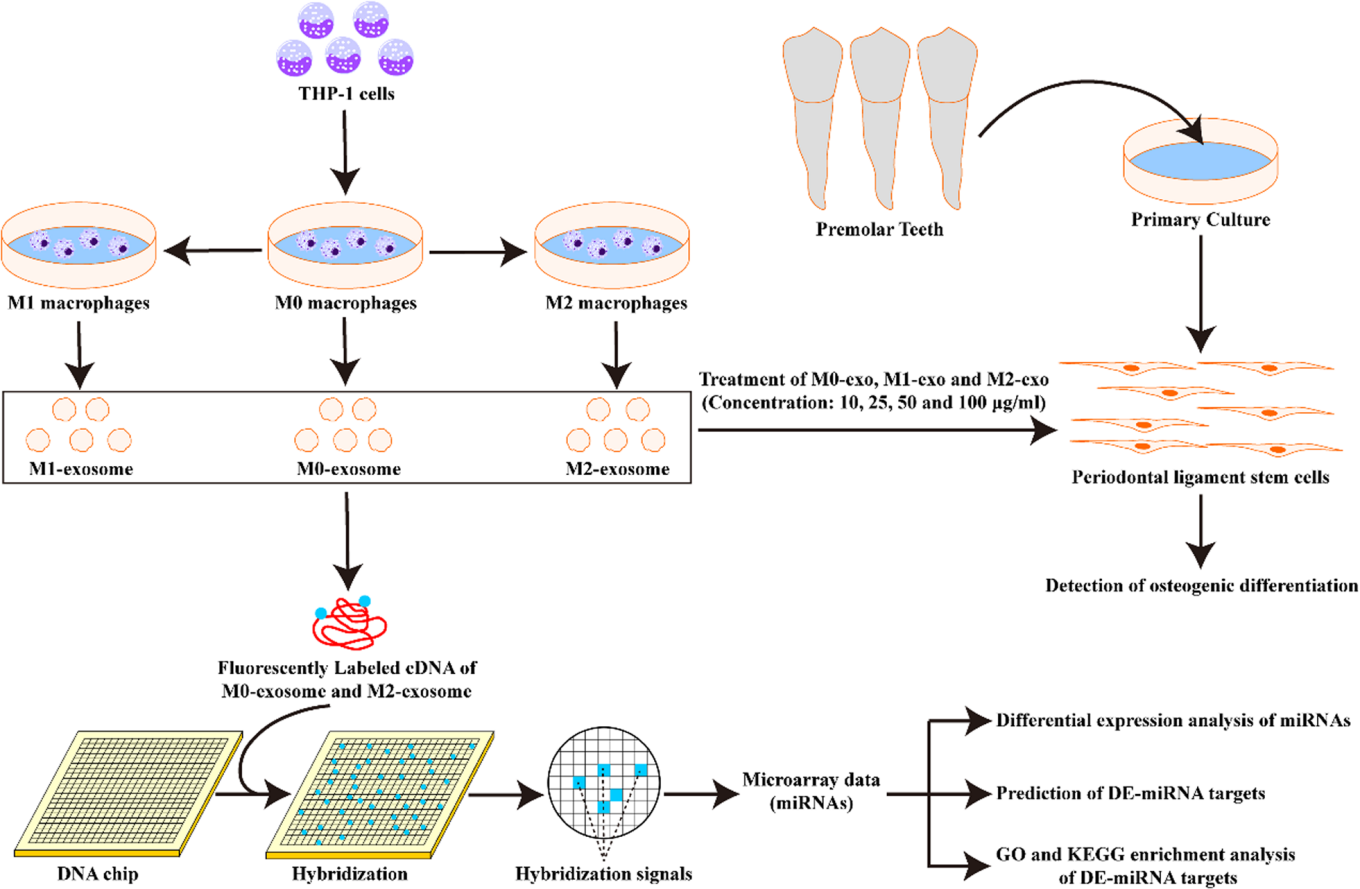
Flow chart of this study

**Fig. 2:**
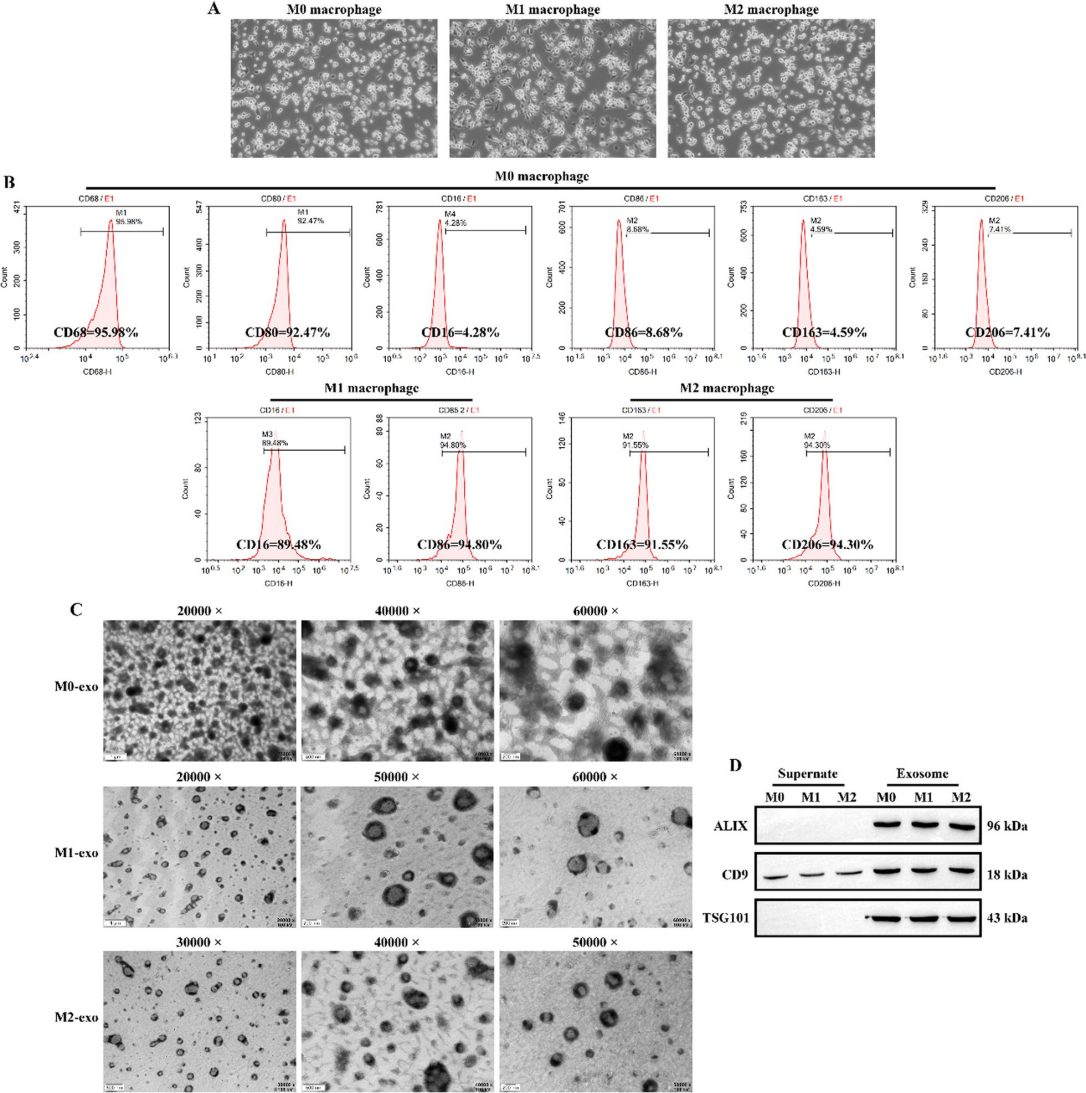
Identification of M0, M1 and M2 macrophage-derived exosomes. **A** Morphology of M0, M1 and M2 macrophages under inverted microscope. Original magnification: 100 × . **B** Flow cytometry identification of the proportion of positive M0, M1 and M2 macrophage markers. **C** Morphology and size of M0-exo, M1-exo and M2-exo under TEM. **D** Western blotting was used to detect the expression of exosomal markers ALIX, CD9 and TSG101 in macrophage supernatants and M0-exo, M1-exo and M2-exo

The flow chart of this study is shown in Fig. 1. As shown in Fig. 2a, after the induction of THP-1 by PMA, the cell morphology appeared to be polygonal with sub-circular shape and transformed into anchorage-dependent cell. After IFN-γ and LPS induction, M1 macrophages long shuttle-shaped cells with elongated pseudopods. After induction of M0 macrophages by IL-4 and IL-13, the morphology of M2 macrophages was similar to that of M0 macrophages and had a shorter pseudopod. Flow cytometry results displayed that the percentage of induced M0 macrophages positive for CD68, CD80 CD16, CD86, CD163 and CD206 were 95.98%, 92.47%, 4.28%, 8.68%, 4.59%, and 7.41%, the percentage of induced M1 macrophages positive for CD16 and CD86 were 89.48% and 94.80%, and the percentage of induced M2 macrophages positive for CD163 and CD206 were > 90%, respectively (Fig. 2b). Therefore, the polarization rates were 85.20% and 86.12% for CD16- and CD86-positive M1 macrophages, and 86.96% and 86.89% for CD163- and 206-positive M1 macrophages. The above results indicate that we successfully induced M0, M1 and M2 macrophages.

Further, we separated M0-exo, M1-exo and M2-exo respectively. TEM revealed that the vesicles isolated from M0, M1 and M2 macrophages were all bilayer structures and ranged in size from 50–150 nm (Fig. 2c). Furthermore, western blotting results demonstrated that M0-exo, M1-exo and M2-exo expressed exosome markers ALIX, CD9 and TSG101, and that ALIX, CD9 and TSG101 were not or less expressed in the cell supernatant (Fig. 2d). The above results illustrate that we have successfully separated M0-exo, M1-exo and M2-exo.

### Identification of hPDLSCs

**Fig. 3:**
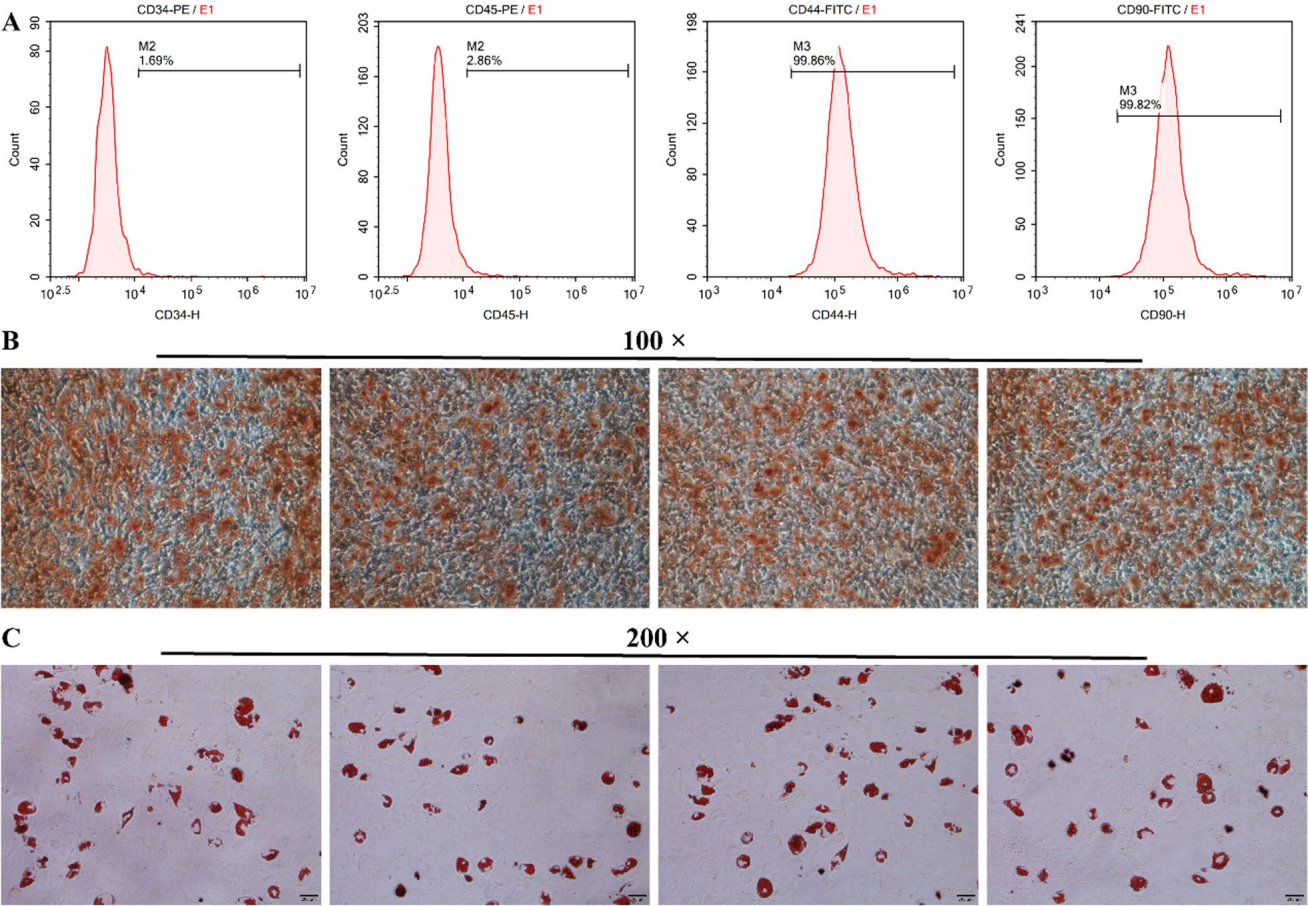
Identification of hPDLSCs. **A** Proportion of positive hPDLSCs markers identified by flow cytometry. **B** ARS and **C** Oil red O staining were performed to assess the osteogenic differentiation and lipogenic differentiation of hPDLSCs, respectively. The four representative images of ARS and Oil red O staining indicate the four replicates of PDLSCs induced osteogenic and lipogenic differentiation, respectively

We cultured hPDLSCs in primary culture and identified the levels of isolated hPDLSCs markers by flow cytometry. The results showed that hPDLSCs were positive for CD44 and CD90 (positive percentage > 90%) and negative for CD34 and CD45 (positive percentage < 5%) (Fig. 3a). Moreover, we assessed the differentiation ability of primary cultured hPDLSCs. As displayed in Fig. 3b-c, primary cultured hPDLSCs had osteogenic and lipogenic differentiation abilities. The above results indicate that we successfully isolated CD34^−^CD45^−^CD44^+^CD90^+^ hPDLSCs.

### Effects of M0-exo, M1-exo and M2-exo on osteogenic differentiation of hPDLSCs

**Fig. 4:**
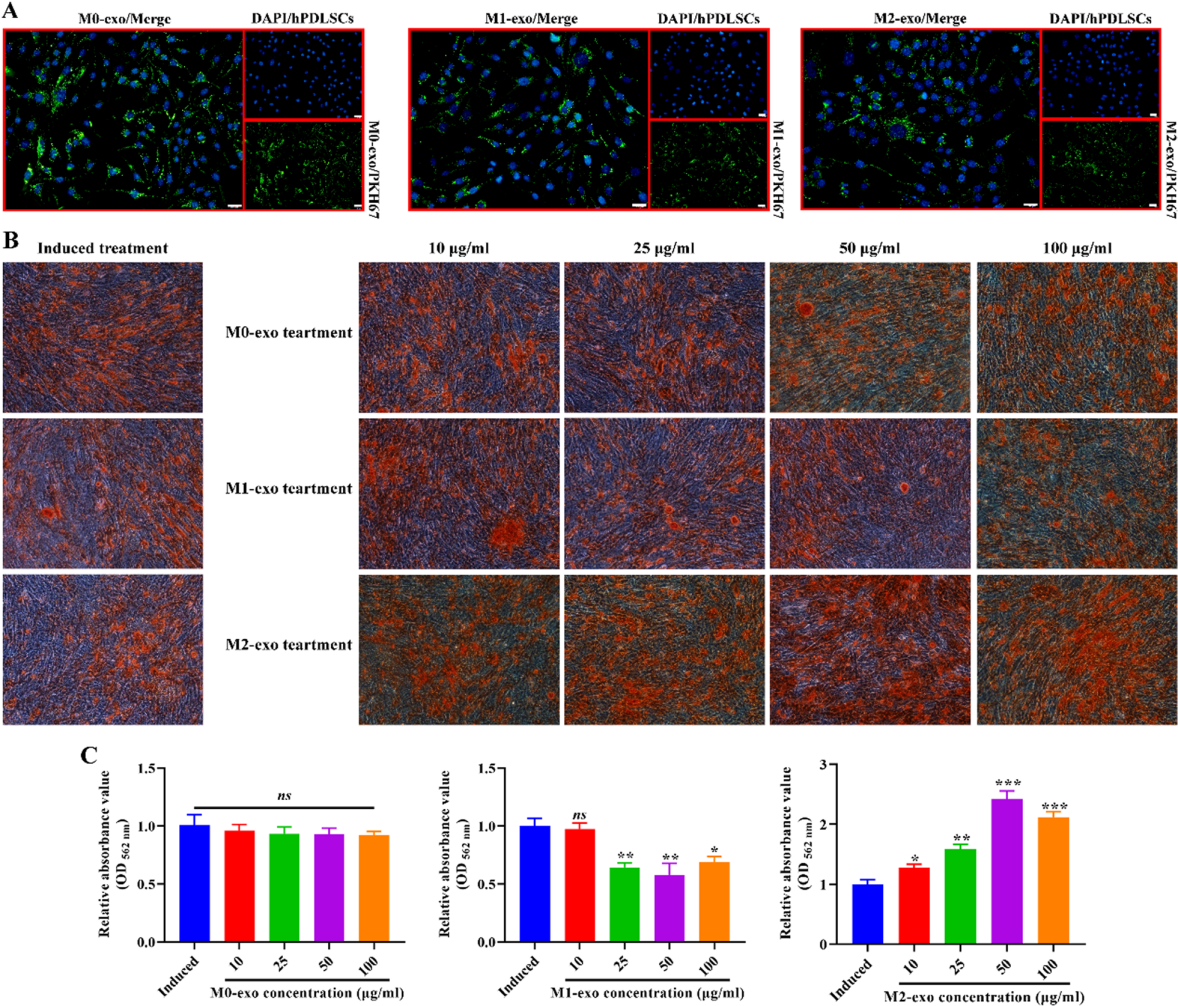
Effect of M0-exo, M1-exo and M2-exo on osteogenic differentiation of hPDLSCs. **A** Fluorescence microscopic observation of exosome uptake by hPDLSCs after PKH67 labeling of M0-exo, M1-exo and M2-exo. Scale bar: 20 μm. **B** The effect of M0-exo, M1-exo and M2-exo on the formation of mineralized nodules was observed by ARS after 21 days of osteogenic induction. Original magnification: 100 × . **C** Spectrophotometric detection of absorbance of mineralized nodules using a spectrophotometer. Values are mean ± SD (n = 3 for each group). Compared to Induced group, *P < 0.05, **P < 0.01, ***P < 0.001, and *ns* means no significant difference

The effects of M0-exo, M1-exo and M2-exo on the osteogenic differentiation of hPDLSCs are not well understood by the available studies. At first, we used PKH67 to label M0-exo, M1-exo, and M2-exo to verify whether hPDLSCs were capable of uptake. The results displayed that M0-exo, M1-exo and M2-exo were all able to enter into hPDLSCs (Fig. 4a). Then, we treated activated macrophages with different concentrations of M0-exo, M1-exo and M2-exo to explore the effects of M0-exo, M1-exo and M2-exo on osteogenic differentiation of hPDLSCs. ARS results exhibited that, under conditions of osteogenic induction, M0-exo had no significant effect on the formation of mineralized nodules, while M1-exo significantly limited the formation of mineralized nodules in hPDLSCs, and M2-exo did the opposite (Fig. 4b-c).

**Fig. 5:**
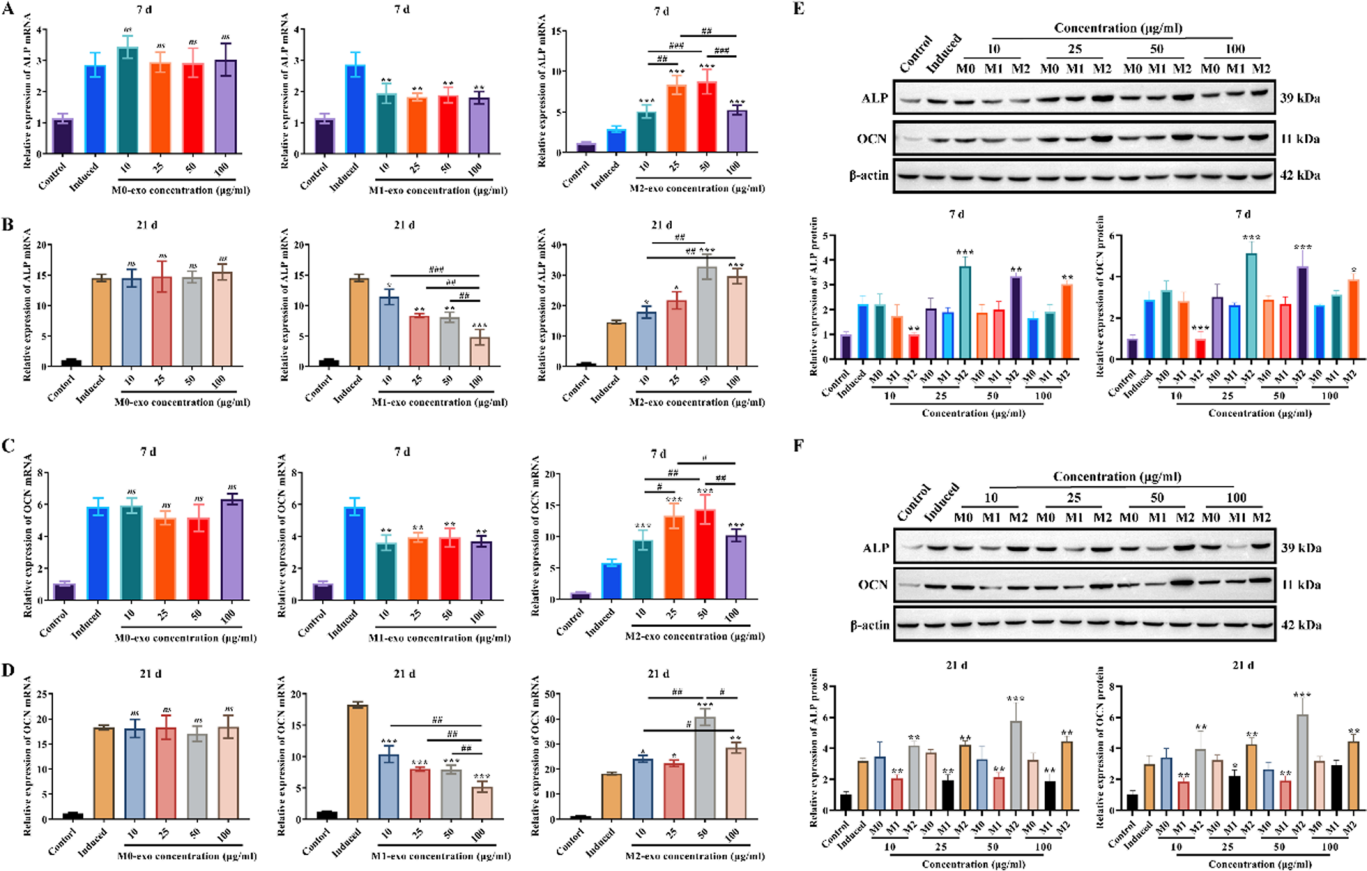
Effect of M0-exo, M1-exo and M2-exo on ALP and OCN expression in hPDLSCs. The expression of (**A**–**B**) ALP and (**C**–**D**) OCN mRNAs were detected by RT-qPCR at day 7 and of osteogenesis induction, respectively. The expression of ALP and OCN protein in hPDLSCs was detected by Western blotting at (**E**) day 7 and (**F**) 21 after osteogenic induction. Values are mean ± SD (n = 3 for each group). Compared to Induced group, *P < 0.05, **P < 0.01, ***P < 0.001; #*P* < 0.05, ##*P* < 0.01, ###*P* < 0.001, and *ns* means no significant difference

RT-qPCR results showed that M0-exo had no significant effect on ALP mRNA expression in hPDLSCs either at day 7 or 21 after osteogenic induction (Fig. 5a–b). Interestingly, M1-exo significantly restrained ALP mRNA expression in hPDLSCs at either the 7th or 21st day, with the most significant effect after 100 μg/ml treatment for the 21st day (Fig. 5a–b). In contrast, M2-exo was able to significantly upregulate ALP mRNA expression, and the effect was most pronounced after 50 μg/ml treatment for 21 d (Fig. 5a–b). OCN mRNA expression was similar to that of ALP (Fig. 5c–d). Furthermore, we explored the effects of M0-exo, M1-exo and M2-exo on the expression of ALP and OCN proteins in hPDLSCs. The results revealed that neither M0-exo nor M1-exo had a significant effect on the expression of ALP and OCN proteins in hPDLSCs at 7 d, but M2-exo at 25 μg/ml, 50 μg/ml and 100 μg/ml upregulated the expression of ALP and OCN proteins in hPDLSCs compared to the Induced group (Fig. 5e). At 21 d of osteogenesis induction, all concentrations of M1-exo reduced ALP and OCN protein expression, while M2-exo did the opposite (Fig. 5f).

The above results demonstrate that M2-exo promotes the osteogenic differentiation of hPDLSCs, while M1-exo facilitates the opposite.

### Identification of DE-miRNA in M0-exo and M2-exo

**Fig. 6:**
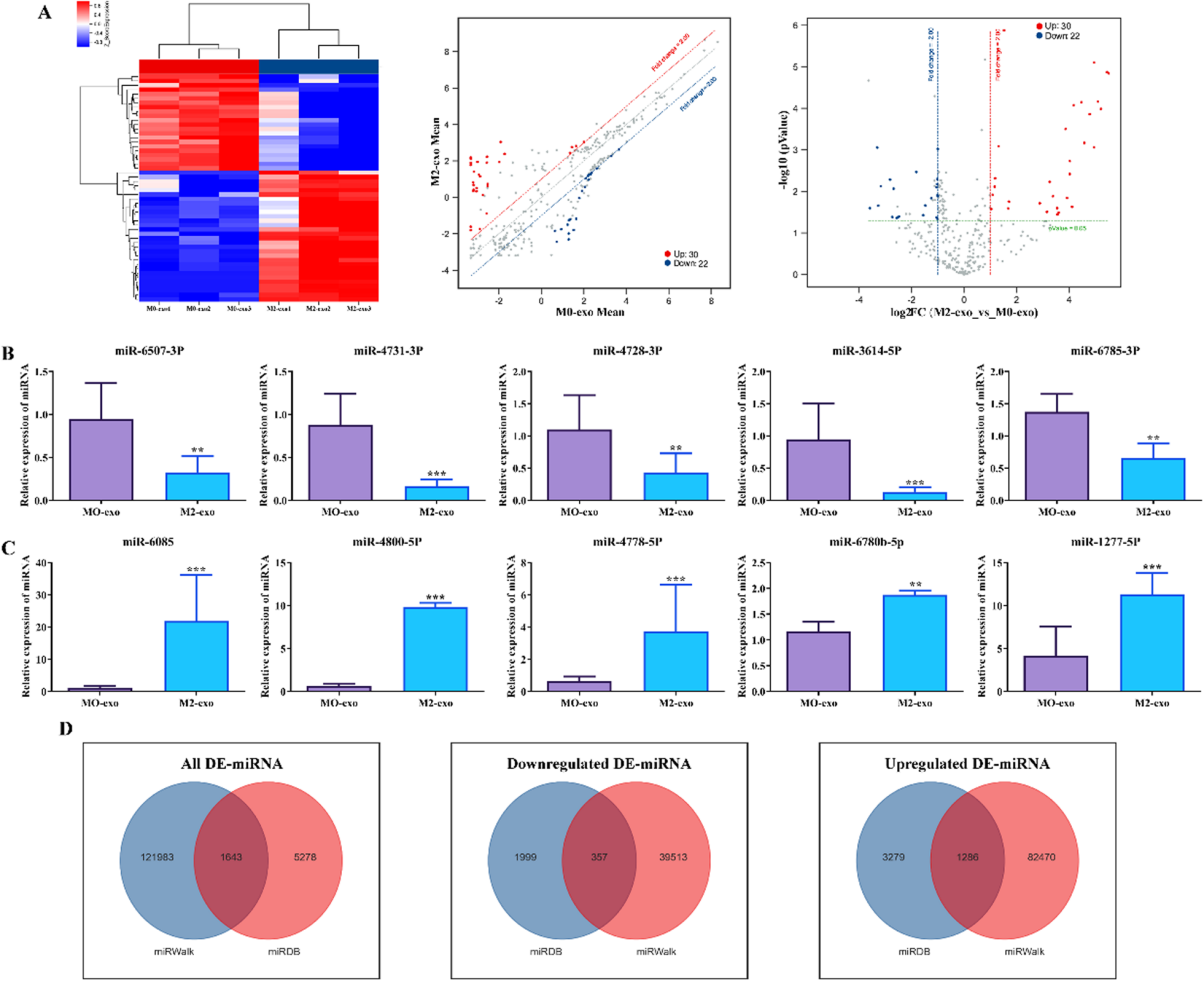
Identification of DE-miRNAs in M0-exo and M2-exo. **A** Differential expression analysis of miRNAs in M0-exo and M2-exo. From left to right are heat map, scatterplot and volcanoplot. Threshold: absolute log2 FC ≥ 1 and *P* < 0.05. RT-qPCR was applied to examine the expression of (**B**) TOP5 down-regulated DE-miRNA and **C** TOP5 up-regulated DE-miRNA in M0-exo and M2-exo. **C** Prediction of target genes for (left) All DE-miRNA, (middle) down DE-miRNA and (right) up DE-miRNA using miRWalk and miRNADB databases. Values are mean ± SD (n = 3 for each group). Compared to M0-exo group, **P < 0.01, ***P < 0.001

As M2-exo can promote osteogenic differentiation of hPDLSCs, while M0-exo has no effect. Consequently, we used miRNA microarray to identify DE-miRNAs in M0-exo and M2-exo. Analysis revealed that a total of 52 DE-miRNAs were present in M2-exo compared to M0-exo, including 22 downregulated and 30 upregulated DE-miRNAs (Fig. 6a). TOP5 of downregulated and upregulated DE-miRNAs are shown in Table 2. To verify this result, we performed RT-qPCR. The results displayed that, compared to M0-exo, the expression of hsa-miR-6507-3p, hsa-miR-4731-3p, hsa-miR-4728-3p, hsa-miR-3614-5p and hsa-miR-6785-3p was significantly downregulated in M2-exo (Fig. 6b), and hsa-miR- 6085, hsa-miR-4800-5p, hsa-miR-4778-5p, hsa-miR-6780b-5p, and hsa-miR-1227-5p were significantly upregulated (Fig. 6c). This result is consistent with the microarray data. In further, we predicted the downstream targets of DE-miRNAs by miRWalk and miRDB databases. The Venn diagram exhibited that all DE-miRNAs had 1643 common targets in both databases, of which downregulated DE-miRNAs had 357 and upregulated DE-miRNAs had 1286 (Fig. 6d).

**Table 2:** TOP5 of downregulated and upregulated DE-miRNA

ProbeID	*P* value	Log_2_ Fold change (FC)	Regulation
hsa-miR-4731-3p	0.0252	− 3.5911	Down
hsa-miR-6785-3p	0.0009	− 3.3081	Down
hsa-miR-6507-3p	0.0220	− 3.2727	Down
hsa-miR-4728-3p	0.0074	− 3.1674	Down
hsa-miR-3614-5p	0.0052	− 2.8212	Down
hsa-miR-6780b-5p	0.0000	5.5106	Up
hsa-miR-4800-5p	0.0000	5.4463	Up
hsa-miR-6085	0.0001	5.2149	Up
hsa-miR-4778-5p	0.0000	5.1013	Up
hsa-miR-1227-5p	0.0000	4.9560	Up

### GO and KEGG enrichment analysis of DE-miRNA targets

**Fig. 7:**
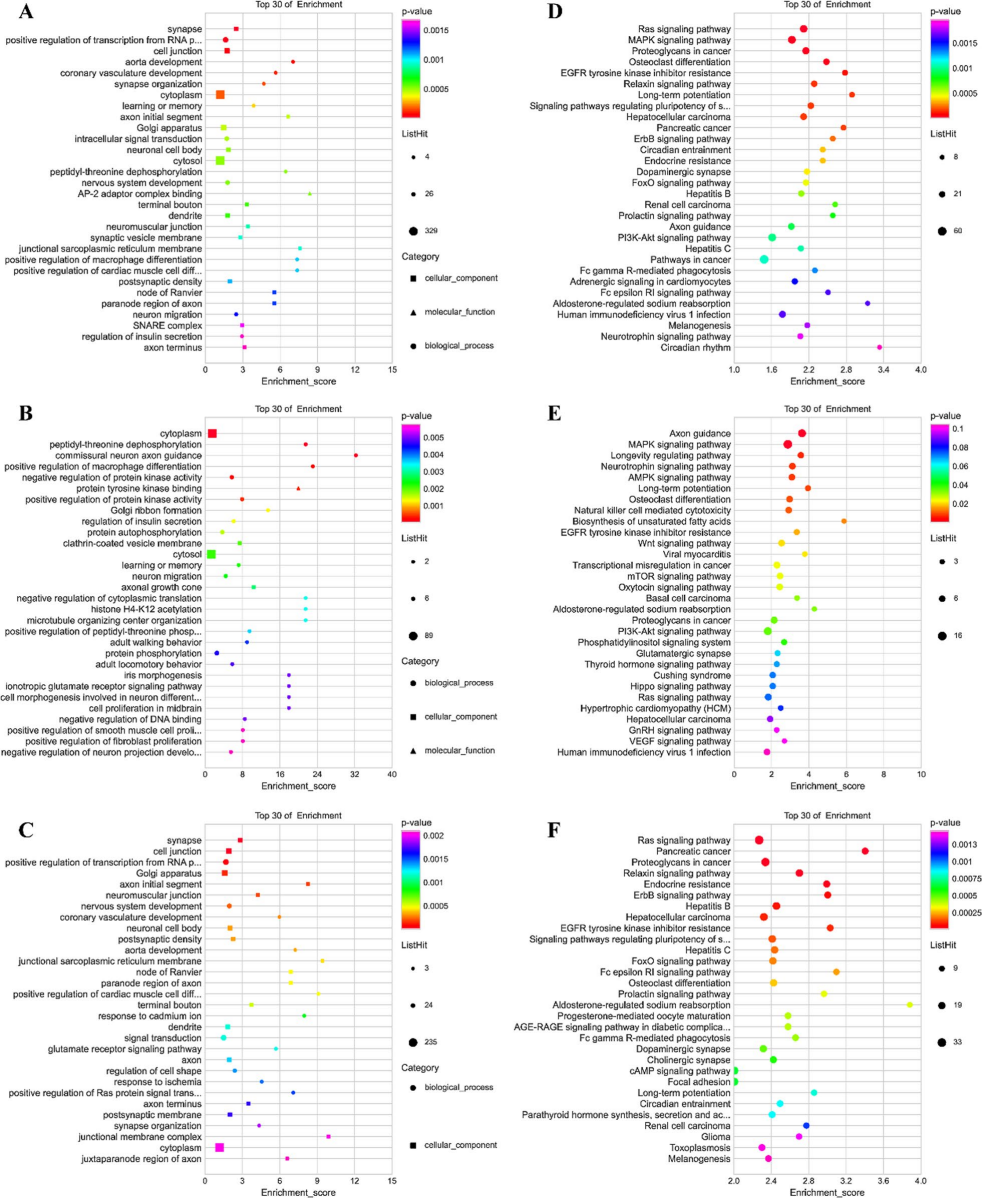
GO and KEGG enrichment analysis of DE-miRNA targets. GO [25] enrichment analysis of (**A**) All DE-miRNA, **B** down-regulated DE-miRNA and **C** up-regulated DE-miRNA target genes were obtained for TOP30 term (*P* < 0.05). The TOP30 pathway was obtained by KEGG [26] pathway enrichment analysis of (**D**) All DE-miRNA, **E** down-regulated DE-miRNA and **F** up-regulated DE-miRNA target genes (*P* < 0.05)

GO enrichment analysis revealed that DE-miRNA downstream targets were enriched for terms including “cell junction”, “neuron migration”, “positive regulation of macrophage differentiation”, “negative regulation of fat cell differentiation”, “negative regulation of osteoblast differentiation”, “osteoclast differentiation”, “neuron differentiation”, which are mainly involved in cell migration and differentiation processes of a variety of cells (Fig. 7a–c and Additional files 1, 2, 3). The results of KEGG enrichment analysis showed that the main enriched pathways of DE-miRNA downstream targets included "Ras signaling pathway", "MAPK signaling pathway", "Osteoclast differentiation", "Th17 cell differentiation", "Th1 and Th2 cell differentiation", " Long-term potentiation" etc. (Fig. 7d–f and Additional files 4, 5, 6). The above results suggest that DE-miRNAs in M2-exo are most likely involved in regulating osteogenic differentiation of hPDLSCs through these targets.

### PPI network construction for DE-miRNA targets

**Fig. 8:**
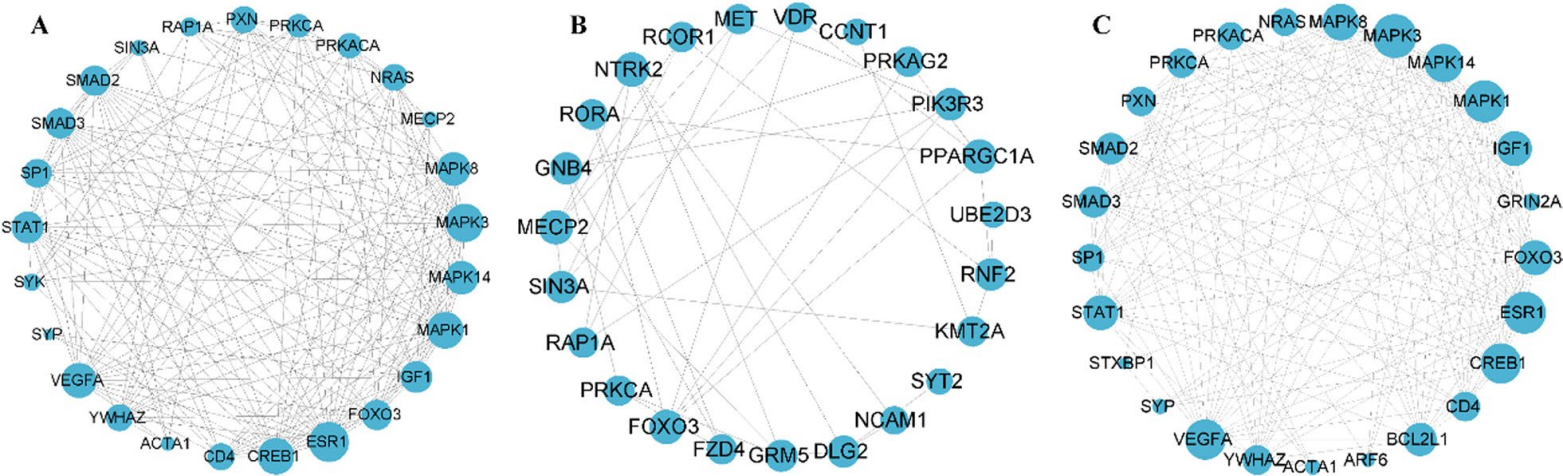
Construction of PPI network for DE-miRNA targets. PPI network of (**A**) All DE-miRNA, **B** down-regulated DE-miRNA and **C** up-regulated DE-miRNA target genes. The top 25 nodes (genes) in terms of connectivity are selected for the graph, and the size of the circle represents the level of connectivity, with larger circles indicating higher connectivity

As displayed in Fig. 8a, the network is a PPI network of all DE-miRNA targets, in which the genes with high linkage include MAPK1/3/8/14, CERB1, ESR1, STAT1, VEGFA, etc. (Additional file 7). The hub gene in the PPI network of downregulated DE-miRNA targets includes MECP2, GNB4, NTRK2, SIN3A, RAP1A, etc. (Fig. 8b and Additional file 8). The PPI network of upregulated DE-miRNA targets was similar to that of all DE-miRNA targets (Fig. 8c and Additional file 9). It is implied that these genes may be key targets of M2-exo involved in regulating osteogenic differentiation of hPDLSCs through the transport of DE-miRNAs.

## Discussion

With the improvement of living standards and the popularization of oral health knowledge, more and more patients are requesting orthodontic treatment to improve facial aesthetics and oral function. However, many patients are often discouraged by the 2–3-year orthodontic cycle and choose to abandon orthodontic treatment or opt for other treatment options. The search for efficient and safe methods to accelerate tooth movement during orthodontic treatment has been one of the hotspot and difficulties in orthodontic research in recent years. To solve this difficulty, the focus is to clarify the biological mechanisms that regulate periodontal tissue remodeling and bone remodeling during OTM. In the present study, we explored for the first time the effects of M0-exo, M1-exo and M2-exo on osteogenic differentiation of hPDLSCs, which provides new insights into the involvement of macrophage polarization in the OTM.

OTM is dependent on the periodontal membrane and the modification of bone tissue, which is a continuous and balanced process [2, 3]. When this process is inadequate or incomplete, it often leads to relapse after orthodontic treatment, which is often encountered in clinical practice. Therefore, the role of the periodontium in supporting the teeth and maintaining periodontal homeostasis is very important for OTM. In the current study, we found that M0-exo, M1-exo, and M2-exo were all taken up by hPDLSCs, and that M2-exo favored osteogenic differentiation of hPDLSCs, while M1-exo showed the opposite, and M0-exo had no significant effect. In fact, there is a lack of studies on the effect of macrophage polarization on the osteogenic differentiation of hPDLSCs. However, the role of macrophage polarization in bone homeostasis of OTM is well documented, with M2 macrophages contributing to the periodontal tissue remodeling process [27–29] and M1 macrophages favoring osteoclastogenesis, periodontal tissue inflammation and bone loss [30–32]. Notably, previous studies have demonstrated that, under normal conditions, PDLSCs promote macrophage polarization toward M2, which facilitates the regeneration of periodontal tissue [15, 33]; In response to lipopolysaccharide or force induction, PDLSCs then increase the ratio of M1/M2 macrophages, which contributes to inflammatory bone remodeling and OTM processes [18, 34]. Obviously, our results complement the interaction between PDLSCs and macrophage polarization, and these findings well compensate for the regulation of osteogenic differentiation of hPDLSCs by macrophage-derived exosomes with different polarization states, and also exposed the intercellular communication between macrophage polarization and hPDLSCs via exosomes during the OTM process. Nevertheless, the mechanisms of M0-exo, M1-exo and M2-exo on osteogenic differentiation of PDLSCs are still unclear. Increasingly, miRNAs have been found to play a role in intercellular signaling through exosomal transport and to be involved in periodontal tissue regeneration and bone remodeling processes. Such as, osteoblast-derived exosome miR-23a-5p can effectively inhibit osteogenic differentiation by targeting Runx2 and promoting YAP1 [35]. M2 macrophage-derived exosome-carried miRNA-26a-5p induces osteogenic differentiation of BMSC [29]. Based on this, we hypothesized that the effect of macrophage-derived exosomes with different polarization states on the osteogenic differentiation of hPDLSCs might be achieved by miRNAs. We found that a total of 52 DE-miRNAs were present in M2-exo macrophages compared to M0-exo, and we verified the expression of TOP5 of downregulated and upregulated DE-miRNAs by RT-qPCR. Remarkably, 52 downstream targets of DE-miRNAs are involved in the differentiation process of multiple cells. Interestingly, a small number of the 52 DE-miRNAs have been shown to have important regulatory roles in osteogenic differentiation. For example, let-7c-5p inhibits the activation of PI3K/Akt signaling pathway by targeting HMGA2, thereby promoting DPSC osteogenic differentiation [36]. Linc02349 as ceRNA activates SMAD5/Dlx5/OSX pathway via sponging miR-25-3p to promote osteogenic differentiation of human umbilical cord-derived stem cells [37]. Emphatically, miR-29 in 52 DE-miRNAs has been shown in OTM to serve as a potential biomarker for periodontal remodeling [38–40]. Notably, the effects of the downstream targets of these DE-miRNAs in the regulation of osteogenic differentiation were also demonstrated, including FOXP1, MAPK14 CSF1, etc. For instance, FOXP1 facilitates osteogenic differentiation of adipose-derived mesenchymal stem cells and bone regeneration in osteoporosis [41]. Phosphorylation of MAPK14 benefits the osteogenic differentiation of human mesenchymal stem cells [42], and inhibits the osteolytic differentiation of bone marrow macrophages [43]. It is strongly indicated that M0-exo, M1-exo and M2-exo are most likely involved in the OTM process by transporting these DE-miRNAs to achieve regulation of osteogenic differentiation of hPDLSCs.

Although the results we obtained are positive, our study still has many flaws. Although we have explored the effect of macrophage-derived exosomes with different polarization states on the osteogenic differentiation of hPDLSCs in vitro, in vivo experiments are lacking for validation. We identified only DE-miRNAs, but their biological functions in the regulation of OTM processes in in vivo and in vitro models remain to be validated. The mechanism of action of DE-miRNA downstream targets is also to be further explored and validated. Moreover, we could also assay the RANKL/OPG ratio in hPDLSCs to further reflect the effect of M0, M1 and M2 macrophages on osteogenesis / osteoclastogenesis. Nevertheless, we elucidated for the first time that differentially polarized macrophage-derived exosomes are regulatory for osteogenic differentiation of hPDLSCs, and provided some potential DE-miRNAs whose functions are also the focus of our subsequent studies.

## Supplementary Information

**Additional file 1**. GO enrichment results for all DE-miRNA targets.

**Additional file 2**. GO enrichment results for down-regulated DE-miRNA targets.

**Additional file 3**. GO enrichment results for up-regulated DE-miRNA targets.

**Additional file 4**. KEGG pathway enrichment results for all DE-miRNA targets.

**Additional file 5**. KEGG pathway enrichment results for down-regulated DE-miRNA targets.

**Additional file 6**. KEGG pathway enrichment results for up-regulated DE-miRNA targets.

**Additional file 7**. Details of the PPI network for all DE-miRNA targets.

**Additional file 8**. Details of the PPI network for down-regulated DE-miRNA targets.

**Additional file 9**. Details of the PPI network for up-regulated DE-miRNA targets.

## Data Availability

The datasets generated and/or analysed during the current study are available in the GEO repository (https://www.ncbi.nlm.nih.gov/geo/query/acc.cgi?acc=GSE209957).
